# CULTURAL ADAPTATION AND RELIABILITY ANALYSIS OF THE EARLY CLINICAL ASSESSMENT OF BALANCE

**DOI:** 10.1590/1984-0462/;2019;37;3;00001

**Published:** 2019-05-09

**Authors:** Ana Paula Bensemann Gontijo, Juliana Maria Pimenta Starling, Graziela Dulce Oliveira, Debora Meier, Marisa Cotta Mancini

**Affiliations:** a Universidade Federal de Minas Gerais, Belo Horizonte, MG, Brazil.; b Hospital Foundation of the State of Minas Gerais, Belo Horizonte, MG, Brazil.

**Keywords:** Cerebral palsy, Postural balance, Child development, Validation studies, Paralisia cerebral, Equilíbrio postural, Desenvolvimento infantil, Estudos de validação

## Abstract

**Objective:**

To translate the Early Clinical Assessment of Balance (ECAB), an assessment scale developed specifically for children and adolescents with cerebral palsy into Brazilian Portuguese, evaluate semantic, idiomatic, experiential and conceptual equivalences, and to examine the face validity and the reliability within and between examiners of the Brazilian version.

**Methods:**

The following steps were done: translation by two independent translators; synthesis of translations; back translation into English; analysis of back-translations by a multidisciplinary committee and the author of the test to develop the final version of the test; test application training; administration of the translated version of ECAB (videotaped) in 60 children and adolescents with cerebral palsy; intra and inter-examiner reliability assessment. Reability was assessed by intraclass correlation coefficient (CCI).

**Results:**

The discrepancies found were related mainly to semantic equivalence and, therefore, there was no need to make cultural adaptations in any of the 13 items on the scale. The rate of agreement was greater than 90% and the reliability of the ECAB-Portuguese total score was excellent both for the intra-rater test (CCI=1.00) and for the inter-rater test (CCI=0.998). Likewise, the reliability evaluation of each of the scale items was also excellent.

**Conclusions:**

The translated version of the ECAB into Portuguese provides a tool for the evaluation of the specific balance for children and adolescents with cerebral palsy with different levels of functioning.

## RESUMO

**Objetivo:**

Traduzir a Early Clinical Assessment of Balance (ECAB), escala de avaliação do equilíbrio desenvolvida especificamente para crianças e adolescentes com paralisia cerebral (PC) para a língua portuguesa do Brasil; avaliar as equivalências semânticas, idiomáticas, experiencial e conceituais; e examinar a validade de face e a confiabilidade intra e interavaliadores da versão brasileira.

**Métodos:**

Estudo envolveu tradução do instrumento por dois tradutores independentes; síntese das traduções; retrotradução para o inglês; análise das retrotraduções por um comitê multidisciplinar; treinamento; administração da versão traduzida da ECAB (gravadas em vídeo) em 60 crianças e adolescentes com PC; e avaliação da confiabilidade intra e interexaminadores. A confiabilidade foi avaliada por meio do coeficiente de correlação intraclasse (CCI).

**Resultados:**

As discrepâncias encontradas foram referentes principalmente à equivalência semântica e, portanto, não houve necessidade da realização de adaptações culturais em nenhum dos 13 itens da escala. A taxa de concordância foi maior que 90%, e a confiabilidade do escore total da ECAB-português foi excelente tanto para o teste intra-avaliador (CCI=1,00) quanto para interavaliadores (CCI=0,998). Da mesma forma, a avaliação da confiabilidade de cada um dos itens da escala também foi excelente.

**Conclusões:**

A versão traduzida da ECAB para o português disponibiliza, para os profissionais da reabilitação infantil, um instrumento confiável de avaliação do equilíbrio específico para crianças e adolescentes com PC com diferentes níveis de funcionalidade.

## INTRODUCTION

Postural control is essential for one to position the body in space, contributing to the maintenance of postural stability and alignment of body segments within the limits of the support base.^1^ ^,^ ^2^ ^,^ ^3^ Children and adolescents with cerebral palsy (CP) present deficiencies in postural control, illustrated by the late development of rectification and balance skills, which compromise functional movement, as well as the transfers between postures and daily life activities.^4^ ^,^ ^5^ Appropriate evaluation procedures allow not only to characterize these children’s postural control profile, but also to monitor the development and follow-up of the effects of rehabilitation programs aimed at this population.

Although CP is a common condition, before the development of the Early Clinical Assessment of Balance (ECAB)^6^ scales, there was no instrument that could assess postural and balance control at all levels of severity of this condition. According to a systematic review performed by Saether et al.,^7^ 22 instruments have been used to assess balance of children and adults with CP. Some of these instruments assess only static balance, while others have been developed for children with low levels of affection. From the demand for an instrument that could assess postural and balance control in children with different levels of severity, McCoy et al. developed ECAB in Canada in 2014 as part of a large project called Move & PLAY (Understanding Determinants of Motor Abilities, Self-Care, and Play of Young Children with Cerebral Palsy).^6^ As this is an instrument aimed at balance evaluation that was recently published, we did not find studies that used it as an instrument to measure outcome.

The use of foreign tests requires a careful process of translation and cultural adaptation, including linguistic issues to guarantee semantic and conceptual equivalence, and may or may not require adaptations of the original version, including rewriting, addition of examples, local normative data, among other edits, in order to make the translated version more relevant and adequate to the socio-cultural specificities of the country where it will be applied.^8^ ^,^ ^9^ ^,^ ^10^ ^,^ ^11^

The process of translation and cultural adaptation of foreign evaluation instruments is common in the area of rehabilitation, considering the existence of some foreign tests of high conceptual and psychometric quality. In addition, the use of validated tools makes it possible to compare and summarize results of scientific investigations, including from different countries, contributing to the advancement of knowledge.^8^ Inview of the above, the objectives of this study were:

- To translate the ECAB into Brazilian Portuguese.
- To evaluate semantic, idiomatic, experiential and conceptual equivalence of the original and translated versions.
- To apply the instrument to a group of children and adolescents with CP to test intra and inter-rater reliability.

The translated version of the instrument will allow to describe and quantify changes in balance skills, which can be used both in clinical practice and scientific research.

## METHOD

ECAB was developed to meet the need of having a measure to assess clinical balance that would encompass postural skills prior to sitting and skills specific to subsequent motor milestones such as standing and walking. The authors had ECAB based on the Movement Assessment of Infants (MAI),^12^ from which seven items were removed, and the Pediatric Balance Scale (PBS),^13^from which six items were removed. ECAB is composed of 13 items subdivided into two parts. Part I evaluates postural control of head and trunk in supine, prone and seated positions in seven items. The answers are graded in four levels of difficulty, with scores from 0 to 3 and maximum total score of 36 points.

Part II has six items evaluating postural control and balance in sitting and standing positions. Scoring is graded in five levels of difficulty, from 0 to 4, but the distance between the values of these five levels is not uniform. In fact, the score of each item was adjusted to the difficulty of the task to be performed: for items 8 and 9, for example, the values were multiplied by 1.5; for items 10 and 11, by 2.5; and for items 12 and 13, by 4 (totaling 64 points at the most). The total evaluation score is 100 points.

The properties of measurement of this instrument have been published and indicate that items in ECAB have high internal consistency (Cronbach’s alpha=0.92) and that construct validity was confirmed by its high correlation with GMFM-66-B & C=0.96 (p<0.001).^6^

ECAB is a low-cost test that does not require specific training, is easy to apply, and whose administration takes approximately 15 minutes.^6^ The test sheet and a video demonstration of its use are available at www.canchild.ca.

The pre-final translated version of ECAB was applied to a sample of 60 children and adolescents diagnosed with CP in different levels of Gross Motor Function System (GMFCS) functional impairment.^14^ The sample size was based on the recommendations by Beaton et al.:^11^ groups of 30 to 40 individuals. Children and adolescents with CP were recruited at the Cavalry Regiment Equine Therapy Center of Alferes Tiradentes (CERCAT), at the Rehabilitation Association of Minas Gerais (AMR), and at private clinics located in BeloHorizonte, MinasGerais.

The inclusion criterion for children and adolescents with CP was to be in physical rehabilitation treatment for at least six months. Age group was not considered inclusion criterion, since the level of functionality is more determinant for acquisition of postural control and balance in children with CP than the age. Children and adolescents with CP who had undergone botulinum toxin application or surgeries prior to six months from the date of test were excluded from the study. Children with other diagnoses associated with CP were also excluded, for example, autism, and children whose cognitive deficits made it difficult to understand the test. Participants were classified according to the severity of motor impairment using GMFCS.^14^

The translation and cultural adaptation of ECAB were authorized by the authors of the original instrument.^6^ Theprocedures of the present study were implemented according to guidelines by Guillemin et al.^8^ and Beaton et al.,^11^ for transcultural adaptation, in compliance with the recommendations by the Scientific Advisory Committee of the Medical Outcomes Trust:

- Initial translation: translation of the instrument into Brazilian Portuguese by two independent translators (T1 and T2), who have Brazilian Portuguese as their mother tongue and have knowledge about the source language of the original version (English), informed of the translation objectives.
- Synthesis of translations: analysis of both translations (T1 and T2) by a group of three researchers with expertise in child rehabilitation, generating a Brazilian Portuguese consensus version (T3).
- Back-translation: back translations of version T3 by two independent translators (R1 and R2), of American origin and fluent in the Portuguese language. Then a comparative analysis of both back-translations was conducted. Divergences identified were classified according to semantic, conceptual, idiomatic or experiential characteristics. This step was also intended to compare the source and final versions, adjusting their equivalences so that discrepancies could be solved. The back-translations, as well asthe list of divergences found, were sent to the author of the test, who made a thorough analysis and gave consent to go ahead with the translation process.
- Committee of experts: approval of the final translated version.

The final translated version was sent to six professionals considered experts in the area of child rehabilitation, so they could evaluate face validity. Finally, the scale was applied to 60children and adolescents with CP for intra and inter-examiner reliability analysis.

The evaluations were carried out from June to December 2015. Prior to data collection, five evaluators studied the Brazilian Portuguese version of ECAB, with each item of the scale being analyzed and any questions subsequently discussed. The videos for evaluation, made available for training at www.canchild.ca, were scored and reviewed. Afterwards, eight children with CP were evaluated using the ECAB, their scores were registered and their results were discussed. The intraclass correlation coefficient (ICC) of all examiners was close to 1. Considering ICC>75 as an excellent result, the evaluators were shown to be reliable and therefore they were able to apply the test. Afterthis training, the process of data collection was started. Parents and children were informed of the evaluation procedures: in which positions the children would be evaluated and which tasks they would have to fulfill. They were also informed about the need for recording so that the reliability of the instrument could be analyzed.

When they agreed to the procedures, they were asked to sign the informed consent form and to fill out a sheet with the child’s data. Children were then evaluated using the ECAB-Brazilian Portuguese. Children in the CP group were evaluated at the site of rehabilitation. Three evaluators participated in intra and inter-examiner reliability assessment. The inter-examiner reliability was made by scoring the videos of children with CP, while intra-examiner reliability was assessed by scoring the same videos after an interval greater than ten days. Thisstudy was approved by the Research Ethics Committee of the Universidade Federal de Minas Gerais (CEP-UFMG), CAAE: 42678815.3.0000.5149.

To describe the participants with CP as per the variables age, sex and GMFCS, frequency, the indexes for central tendency (mean) and dispersion (standard deviation - SD) were used.

Intra and inter-examiner reliability were tested by using the final version of ECAB (Brazilian Portuguese). Reliability was assessed considering the individual scores of all 13 items making up the scale and the total evaluation score. For intra and inter-examiner reliability, videos of the 60 participants were analyzed and scored in two different moments, with intervals never less than 10 days. ICC was used to assess reliability and the following classification was adopted: weak agreement, ICC<0.40; moderate, ICC≤0.75; and excellent, ICC>0.75.^15^

To analyze face validity, six professionals from the field of child rehabilitation and with great clinical and research experience were invited. Face validity indicates whether the instrument appears to be appropriate for the purpose of the study and field of knowledge.^16^This property evaluates usability, wording, overall style, format consistency and clarity of the instrument, according to professionals who are potential users.^16^ ^,^ ^17^ Thesesix professionals were invited to answer questions to:

- the clarity of wording of items;
- the pertinence of each item to evaluate postural control and balance;
- the ability to perform test items of the target population.

The evaluation was made based on a Likert scale to sort answers in five scores (1 **-** strongly disagree, 2 **-** disagree, 3**-** maybe, 4 **-** agree, and 5 **-** strongly agree). The frequency of agreement was then calculated.

Statistical Package for the Social Sciences (SPSS) version 21.0 (SPSS Statistics for Windows, Armonk, NY, USA) was used for all analyzes, with significance level α=0.05 and 95% confidence interval (95%CI).

## RESULTS

In the process of translation of ECAB, from the two back-translations (R1 and R2) of the Brazilian Portuguese version (T3), 55 discrepancies between target and original versions were identified. Of these, 33 were related to semantic equivalence (i.e., when the two translations have similar meanings); 21 related to idioms (i.e., when there is difficulty in translating colloquial expressions of a particular language, with consequent need to replace words with terms that are more recurring in the language); and one related to conceptual equivalence (i.e., when the conceptual meaning of the word varies between cultures).

Regarding the discrepancies related to semantic equivalence, words like “start” and “begin” were translated as “*inicie*”; “if indicated” and “if so directed”, as “*se indicado*”; and “trials” and “attempts” as “*tentativas*”.

As for idiom equivalence, the researchers chose to translate the term “full points”, which in a literal translation would be “pontos cheios”, by “*pontuação total*” (full score), since it is a more common term in Brazil. The expression “chin-tuck”, whose literal translation would be “*dobrar/aconchegar o queixo no peito*”, was translated as “*tocar o queixo no peito*” (touch the chin in the chest). However, the committee of experts taking part in the translation process decided to keep the expression chin-tuck, followed by the explanation of “touching the chin in the chest”, as this is an expression widely used in Brazilian literature by professionals of rehabilitation.

Regarding conceptual equivalence, the direct translation of the word “early” would be “*precoce*” (precocious), but in Brazilian Portuguese this word refers to something accomplished before the correct time. At first, the committee of experts decided to use “*inicial*” (initial).

The 55 discrepancies found and the two back-translations were sent to the author of the test who, after analysis, requested only one modification for the only divergence found of conceptual equivalence. The name of ECAB assessment was initially translated as “*Avaliação Clínica Inicial do Equilíbrio*” (Initial Clinical Assessment of Balance). The author asked that the term “precoce” were used instead of “*inicial*”, which was accepted by the group of researchers. The name of the evaluation into Brazilian Portuguese became, therefore, “*Avaliação Clínica Precoce do Equilíbrio*” (Early Clinical Evaluation of Balance) (ECAB-Brazilian Portuguese).

The group of children with CP had 38 (63.3%) male participants, with age ranging from one to 12 years old (mean=7.17years, SD=2.80). As for GMFCS, 14 (23.3%) children were classified as GMFCS I; 12 (20%) as GMFCS II; eight (13.3%) as GMFCS III; 15 (25%) as GMFCS IV; and 11 (18.3%) as GMFCS V. Considering the topography of the affected limbs, most children presented bilateral involvement (18 (30%) with spastic quadriplegia and 19 (31.7%) with spastic diplegia), while a smaller number of participants had unilateral involvement (10 (16.6%) with spastic hemiplegia). In addition, nine (15%) children had ataxia and four (6.67%) had hypotonia.

Inter-examiner reliability was analyzed in each item of the scale and for the total score. The inter-examiner reliability was excellent when analyzing each item individually (ICC>0.90) and for the total score (Table 1). Total score intra-examiner reliability was ICC=1.00 (Table 2). The frequency of agreement of experts regarding the clarity of writing, the pertinence and capacity of the target population to go through the items of ECAB-Brazilian Portuguese was higher than 90% (Table 3).

**Table 1:** Inter-examiner reliability of partial and total scores of the Early Clinical Assessment of Balance, Brazilian Portuguese version, in children with cerebral palsy.

Itens in ECAB	ICC	95%CI
ECAB 1a - Retificação da cabeça - lateral (esquerdo)	0.978	0.965-0.987
ECAB 1b - Retificação da cabeça - lateral (direito)	0.985	0.976-0.990
ECAB 2 - Retificação da cabeça - extensão	0.974	0.85
ECAB 3 - Retificação da cabeça - flexão	0.958	0.933-0.975
ECAB 4a - Rotação de tronco (esquerdo)	0.989	0.982-0.993
ECAB 4b - Rotação de tronco (direito)	0.993	0.990-0.996
ECAB 5a - Reações de equilíbrio na postura sentada (esquerdo)	0.948	0.917-0.969
ECAB 5b - Reações de equilíbrio na postura sentada (direito)	0.961	0.938-0.977
ECAB 6a - Extensão protetora - lado (esquerdo)	0.951	0.922-0.971
ECAB 6b - Extensão protetora - lado (direito)	0.976	0.962-0.986
ECAB 7a - Extensão protetora - para trás (esquerdo)	0.953	0.925-0.972
ECAB 7b - Extensão protetora - para trás (direito)	0.957	0.932-0.974
ECAB 8 - Sentado sem apoio nas costas e com os pés apoiados no chão ou em um banco	0.996	0.994-0.998
ECAB 9 - De sentado para de pé	0.992	0.987-0.995
ECAB 10 - Em pé sem apoio com os olhos fechados	0.998	0.996-0.998
ECAB 11 - Em pé, sem apoio, pés juntos	0.995	0.992-0.997
ECAB 12 - Gira 360 graus	0.998	0.996-0.999
ECAB 13 - Colocando os pés de maneira alternada em um degrau enquanto em pé sem apoio	0.986	0.977-0.991
TOTAL	0.998	0.997-0.999

ECAB: Early Clinical Assessment of Balance; ICC: intraclass correlation coefficient; 95%CI: 95% confidence interval.

**Table 2:** Intra-examiner reliability of total score for the Early Clinical Assessment of Balance for Portuguese of Brazil in children with cerebral palsy.

**Assessment 1 *versus* Assessment 2**	ICC (95%CI)*
Examiner 1	1.00*
Examiner 2	1.00*
Examiner 3	1.00*

ICC: intraclass correlation coefficient; 95%CI: 95% confidence interval; *ICC = 1.00 assigned to items with 100% agreement between examiners.

**Table 3:** Frequency of agreement of experts regarding the clarity of the essay and the pertinence and capacity of the target population to understand and perform the items of the Early Clinical Assessment of Balance for Brazilian Portuguese.*

	Strongly disagree	Agree	Maybe	Agree	Strongly agree
Clarity	0	0	0	18 (23.1)	60 (76.9)
Pertinence	0	0	1 (1.3)	12 (15.4)	65 (83.3)
Capacity to perform the item	0	0	3 (3.8)	12 (15.4)	63 (80.8)

*Values expressed as n (%).

## DISCUSSION

The purpose of this study was to perform the cultural adaptation and analysis of face validity and reliability of ECAB for Brazilian Portuguese. Given the scarcity of instruments to assess balance for ​​child rehabilitation developed specifically for the Brazilian population, this study provides the translated and culturally adapted version of ECAB balance test into Brazilian Portuguese. ECAB is the first instrument that assesses postural and balance control developed specifically for children with CP of different functional levels (GMFCS I to V). The translated version will make it possible to document gains derived from therapeutic interventions, as well as to contribute to clinical decision-making and to be used in research in this population. The methodology used followed the guidelines of the literature as to guarantee an appropriate version when it comes to semantic and cultural aspects

In the translation, the small changes made, according to suggestions by the multidisciplinary committee, added greater understanding of items in the instrument. The only modification requested by the author of the test was readily met. Sincethe events evaluated were not different from the concepts of the target culture, there was no need for modifications in this matter.

Regarding face validity of ECAB, the opinion of experts indicates that the items are clear and pertinent for the evaluation of postural control and balance. There was also a high frequency of agreement between professionals about the ability of children and adolescents with CP at all levels of GMFCS to perform the test.

Considering inter-examiner reliability, the ICC was 0.998 and corroborates with the ICCs between examiners of the original instrument, which was 0.989 (0.98-0.99).^6^When the 13 items were analyzed separately, ICC were also considered excellent. The high inter-examiner ICC found in this study can be attributed to the extensive theoretical-practical training to which the evaluators were submitted.

Intra-examiner reliability was also excellent, indicating 100% agreement, similar to the indexes found by Randall etal.,^18^ which was 0.987 (CI=0.97-0.99). Despite the similarity in indices, the procedures used to evaluate intra-examiner reliability were very specific. While in this study the examiners scored the videos at two distinct times, with intervals never less than 10 days after the first scoring; in the study by Randall etal.,^18^ the examiners reassessed the children at intervals of up to two weeks. The consistency in the magnitude of reliability indices of this study and that of Randall et al.^18^suggests that the intra-examiner stability of ECAB seems to be robust and not influenced by different reliability assessment procedures.

The instruments available in the literature evaluate different aspects of balance in children and adolescents with CP, and some of them are restricted to the level of severity of the children being evaluated.^7^These include PBS,^13^ already translated into Brazilian Portuguese,^19^and the Pediatric Reach Test (PRT).^20^ When applied to children with CP, PBS is restricted to those classified only at GMFCS levels I or II (i.e., mild impairment). This restriction results from PBS assessing the balance in activities that require the child, at first, to be able to stand. On the other hand, while PRT may be applied to children with CP of all skill levels, it is restricted to assessing balance response to an internal disturbance, i.e. reaching the range. Different from the previously mentioned instruments, ECAB evaluates postural control in both static and dynamic situations and can be used in children of all levels of GMFCS. Furthermore, and according to Randall et al.^18^, although both tests (PRT and ECAB) show strong psychometric properties, ECAB has higher reliability and validity indexes compared to PRT, with fewer measurement errors and better potential to detect changes over time.

Therefore, as this translation of ECAB is the first validated instrument for the assessment of balance in children with CPs of different levels of functionality, its relevance to child rehabilitation is huge. The results of the translation process, the high level of agreement between professionals in relation to clarity and pertinence of items, and the high reliability indices inter- and intra-examiners found in this study, provide clinicians and researchers with an instrument to assess balance of easy applicability and low cost for the Brazilian pediatric population diagnosed with CP.

Conclusion is that the transcultural adaptation of ECAB into Brazilian Portuguese was performed according to guidelines recommended in the literature and proved to be a reliable instrument to assess postural and balance control in children with CP of different levels of functionality. Brazilian professionals of child rehabilitation will have access to an instrument that, in addition to providing information about the path of developing children’s balance with CP according to each GMFCS level, will disclose the effectiveness of interventions directed to these outcomes.
